# Standard inocula preparations reduce the bacterial diversity and reliability of regulatory biodegradation tests

**DOI:** 10.1007/s11356-013-2064-4

**Published:** 2013-09-17

**Authors:** Andrew K. Goodhead, Ian M. Head, Jason R. Snape, Russell J. Davenport

**Affiliations:** 1 Sartorius Mechatronics UK Limited, Longmead Business Centre, Blenheim Road, Epsom, Surrey KT19 9QQ UK; 2 School of Civil Engineering and Geosciences, Newcastle University, Cassie Building, Newcastle upon Tyne, NE1 7RU UK; 3 Brixham Environmental Laboratory, AstraZeneca UK Ltd., Freshwater Quarry, Brixham, Devon TQ5 8BA UK

**Keywords:** Biodegradation, Persistence, Diversity, Bacterial community structure, 4-Nitrophenol, REACH

## Abstract

OECD ready biodegradability tests have been central to understanding the biodegradation of chemicals from a regulatory perspective for many decades. They are not fit for contemporary prioritisation of chemicals based on persistence, however, due to the low concentration of inocula used, short duration and high variability between tests. Two OECD standard inoculum pretreatment methods (settlement and filtration) were investigated to observe their effect on the probability of biodegradation and associated changes in bacterial community structure and diversity of inocula sourced from the activated sludge process of wastewater treatment plants. Both settlement and filtration were shown to dramatically and significantly reduce the probability and increase the variability of biodegradation of 4-nitrophenol compared to the use of unprocessed inocula. These differences were associated with a significant hundred-fold reduction in cell numbers and solids content and a significant shift in bacterial community structure that was sometimes accompanied by significant reductions in detectable operational taxonomic unit richness and evenness. The natural variation (between different environments) and variation due to differential selection of bacterial communities (by different pretreatment methods) is offered as an explanation for the historical high variability in standard OECD ready biodegradability tests.

**Electronic supplementary material:**

The online version of this article (doi:10.1007/s11356-013-2064-4) contains supplementary material, which is available to authorized users.

## Introduction

Since the advent of modern industrial chemistry in the 1860s, chemicals have helped to improve the lives, well-being, wealth and productivity of societies around the world. Total annual global chemical production has risen from 1 million to over 400 million tonnes in the last 80 years (Motaal 2009), and 100,204 chemicals are currently registered in the EU alone, including those used in everyday products such as pharmaceuticals, personal care, domestic cleaning and hygiene products (European Commission JRC, Institute for Health and Consumer Protection 1990). Recent estimates indicate that hazard information for up to 90 % of these chemicals is lacking, a proportion of which may have carcinogenic, mutagenic and developmental effects on wildlife or humans (Binetti et al. 2008). Concerns during the past decade that the pre-existing chemical regulations were not protective enough led to a recent shift in regulatory emphasis towards the identification of chemicals with persistent, bioaccumulatory and toxic (PBT) properties, and prioritising the collection of data for those chemicals with high production volumes (e.g. Registration, Evaluation, Authorisation and Restriction of Chemicals, REACH; Toxic Substances Control Act, TSCA; Canadian Environmental Protection Act, CEPA). Regulation is exerted through identification of hazards and the assessment of risks that manufactured chemicals pose to human health and the environment. These assessments are based on estimated emissions of chemicals and their likely concentrations in the environment compared to the likelihood of exposure and harm done by the chemicals. The assessments invariably require information on the fate, ecotoxicology and toxicology of the chemicals often determined by a battery of internationally recognised standardised tests.

Biodegradation is an important but poorly understood fate process that plays a central role in chemicals’ ability to persist and remain unchanged in the environment and in determining their eventual environmental concentrations and thus their likelihood of presenting an exposure risk (Hertwich et al. 1999; Huijbregts et al. 2003). Information on biodegradability of chemicals is required for hazard classification (e.g. classification, labelling and packaging), environmental risk assessment (chemical safety assessment) and persistency assessments (e.g. PBT; very persistent and very bioaccumulative, vPvB) (ECHA 2008). The testing paradigm for biodegradability, exemplified by the OECD guidelines (OECD 1992a), involves a series of tiered tests whereby the stringency of the test decreases while the complexity and realism of the test increases. This testing paradigm was originally implemented to identify those chemicals that would undergo rapid ultimate biodegradation (ready biodegradability) or have the potential to be degraded (inherent biodegradability).

The ready biodegradability test (or RBT, e.g. OECD 301 tests) (OECD 1992b) is a first tier screening test that has been the central foundation for understanding the biodegradation of chemicals in regulatory frameworks for nearly three decades. This can be attributed to their relative low cost, ease of interpretation and the amount of data already collected for chemicals since the introduction of RBTs in the 1970s (Aronson et al. 2006). RBTs are highly prescribed, standardised and conservative regulatory tests that measure the relative biodegradability of chemicals (e.g. OECD 1992b). A central assumption in all biodegradability tests is that the diversity present in a randomly selected sample from a natural microbial population provides the requisite range of microorganisms for assessing biodegradation potential of a particular compound in any given environment. They rely on the potential of an arbitrary inoculum of a mixed microbial population at low concentration to degrade a chemical that is provided as a sole organic carbon source, over a 28-day period under standardised laboratory conditions in a buffered basal salts medium. While the sampling and preparation of inocula is highly prescribed, the source, type of preparation and concentration of test inocula can vary widely between tests (Table 1). If biodegradation does occur, it is generally supposed that the bacteria responsible are minority organisms with a minimum abundance of just one in the initial inoculum. By using inocula of low density in a relatively low test volume, the probability of minority organisms being present, even in replicate samples, is reduced and their inclusion is thus a matter of chance (e.g. Thouand et al. 1995; Ingerslev and Nyholm 2000). In addition, the diversity of microorganisms in such inocula will be relatively low in comparison to the original sample and its source environment as the total number of individuals will be drastically reduced, a rationale that is formally captured in ecology, rarefaction curves (Sanders 1968), species–area relationships (Arrhenius 1921) and island biogeography (MacArthur and Wilson 1967). Some of the differences between tests have been attributed to the ‘inoculum quality’ (Blok and Booy 1984), which refers to differences in the microbial community between sources. Consequently, RBTs suffer from a large degree of variation both within and between different studies on the same chemical (Blok and Booy 1984). For example, 4-nitrophenol has been shown to produce variable RBT outcomes with different inocula (Painter 1995; Nyholm and Preben 1992; Nyholm et al. 1984; Thouand et al. 1995; Gerike and Fischer 1979; Van Ginkel et al. 1995). Some studies have even shown the complete lack of 4-nitrophenol biodegradation using an 18-day acclimated inoculum (Boatman et al. 1986).

**Table 1:** Typical solids, effluent and bacterial cell concentrations in OECD recommended RBTs (modified from OECD 1992a, b)

OECD test designation	301 A	301 B	301 C	301 D	301 E	301 F
Name	DOC die away	CO_2_ evolution	MITI (I)	Closed bottle	Modified OECD screening	Manometric respirometry
mg/l SS	≤30	≤30	30	n/a	n/a	≤30
ml effluent added/l	≤1	≤1	n/a	≤0.05	0.05	≤1
Approx. cells/ml	10^4^–10^5^	10^4^–10^5^	10^4^–10^5^	10^1^–10^3^	10^2^	10^4^–10^5^
Source of inoculum						
1. Activated sludge	✓	✓	✓			✓
2. Secondary effluent	✓	✓	✓	✓	✓	✓
3. Surface water	✓	✓	✓	✓	✓	✓
4. Soil	✓	✓	✓			
5. Mixture of above	✓	✓	✓			✓
Pretreatment options						
1. Settling for 30 min or 1 h	✓	✓	✓	✓	✓	✓
2. Filtering through fine sieve	✓	✓				✓
3. Filter through a coarse filter paper	✓	✓		✓	✓	✓
4. Precondition^a^	✓	✓		✓	✓	✓
5. Preculture with glucose/peptone			✓			

*n/a* not applicable

^a^Pre-conditioning consists of aerating the inoculum (in mineral medium) for 5–7 days at the test temperature but does not allow pre-adaptation to the test substance

Efforts to investigate this challenging problem to date have been hampered by a lack of suitable methods and theory to assess the diversity of these inocula in relation to biodegradation performance. In the last 15 years, there has been a revolution in molecular DNA-based methods to measure diversity (Head et al. 1998), but there have been few previous reports of their use to assess chemical regulatory biodegradation tests (Forney et al. 2001).

This study was undertaken to resolve how the international ‘standard’ OECD guidelines for inocula preparations (OECD 1992b) may alter the detectable bacterial diversity compared to the original environmental samples from which they were prepared. In addition, we assessed the associated effects that these preparation methods have on the probability or reliability of observing biodegradation. Miniaturised RBTs were performed, using 96-well microplates (Thouand et al. 1995), to increase throughput and derive more statistically robust outcomes. 4-Nitrophenol was chosen as the test chemical as it has been extensively studied with respect to biodegradation and has also been previously used in miniaturised RBTs (Thouand et al. 1995).

## Materials and methods

### Sample collection and inocula preparation

Inocula were derived from activated sludge sampled from the aeration lanes of Tudhoe Mill and Sedgeletch domestic wastewater treatment plants (WWTP) in the north-east of England. The sample sites were chosen for the difference in their sludge age (Table 2), as longer sludge ages would infer greater bacterial diversity (Akarsubasi et al. 2009). Activated sludge was sampled using sterile equipment and kept aerobic during transportation to the laboratory. The sludge was immediately prepared in the following ways: (i) left to settle for a total of 1 h, at which point the supernatant was drawn off to be used as an inoculum (OECD A and B, D–F) (OECD 1992b); (ii) filtered through a 5-μm nylon cloth (Normesh, Oldham) (Thouand et al. 1995) to remove mineral clumps and protozoa, with collection of the filtrate as an inoculum (OECD A and B, D–F) (Thouand et al. 1995; OECD 1992b); and (iii) washed once, centrifuged and resuspended in mineral media and then used directly as an inoculum (unprocessed activated sludge). For the settled supernatant samples (method i above), a range of inocula concentrations were produced by diluting a known volume (0.01, 0.1, 0.5, 1, 2, 3, 4, 5 and 100 ml) of pretreated sample in up to 300 ml of sterile mineral medium in sterile BOD bottles (OECD D and E) (OECD 1992b). Samples that underwent filtration (method ii above) were serially diluted tenfold to 10^−4^. Following determination of total cell counts (see below), inocula from different treatment plants were normalised to the same cell concentration (10^8^ cells/ml), and subsequently tenfold serial dilutions spanning up to 5 orders of magnitude were used to inoculate high-throughput biodegradation screening tests.

**Table 2:** Characteristics of the activated sludge sampled from Tudhoe Mill and Sedgeletch wastewater treatment plants before and after pretreatment by filtration for inocula preparation according to international guidelines (OECD 1992a, b)

WWTP	Sample	Sludge age (days)	Total suspended solids (TSS mg/l)	Total cell count (TCC × 10^7^/ml)^a^	4-Nitrophenol degraders (×10^3^/ml)^a^	Mean OTU richness^a^	Mean evenness^a^
Tudhoe Mill	Activated sludge	18	3,750	169 ± 92	0.44 ± 0.06	27.8 ± 4.4	0.72 ± 0.07
	Filtrate	n/a	76.5	1.63 ± 0.36	n.d.	22.2 ± 6.4	0.58 ± 0.06
Sedgeletch	Activated sludge	8	3,250	801 ± 0.05	57.99 ± 0.06	19.8 ± 3.1	0.62 ± 0.04
	Filtrate	n/a	12	14.7 ± 1.91	n.d.	16.2 ± 6.6	0.62 ± 0.04

*n.d.* not detected

^a^Values ± standard deviation

### High-throughput biodegradation screening tests

Miniature high-throughput biodegradation screening tests (HT-BSTs) for 4-nitrophenol were used to mimic RBTs as generally described by Thouand et al. (1995) with slight modifications (see below). These tests mimic international standard OECD RBTs with respect to the inocula, mineral media and controls used, but have much higher replication.

HT-BSTs were carried out using presterilised 96-well plates (BD Falcon) containing 250 μl of sterile 4-nitrophenol medium. The 4-nitrophenol medium contained the following (per litre): 4-nitrophenol (Sigma) at a final concentration of 10 mg carbon (C) l^−1^, NH_4_Cl with a final C/N ratio of 10, 1.4 KH_2_PO_4_ and 1.5 K_2_HPO_4_ (by mass). The medium was adjusted to pH 7 using KOH. This was then sterilised by passing through a 0.22-μm syringe filter (PALL, Ann Arbor, USA). To each well, 50 μl of the diluted or undiluted inoculum was added. One 96-well plate was used per dilution. Plates were then wrapped in Parafilm (Pechiney Plastic Packaging Company, USA) and placed in a moisture-saturated chamber (mineral media on blotting paper) to prevent evaporation. The plates were placed in an incubator at 30 °C for 28 days (Thouand et al. 1995; OECD 1992b). 4-Nitrophenol decolourises as it degrades, and so after the test period, colourless wells were counted at each dilution in order to calculate the probability (frequency) of the biodegradation of 4-nitrophenol and the specific number of 4-nitrophenol degraders was calculated using the most probable number (MPN) approach using the following formula (APHA 2005):

1 $$ \mathrm{MPN}/\mathrm{ml}=\frac{\mathrm{No}.\kern0.5em \mathrm{of}\kern0.5em \mathrm{positive}\kern0.5em \mathrm{tubes}}{\sqrt{\left(\mathrm{ml}\kern0.5em \mathrm{sample}\kern0.5em \mathrm{in}\ \mathrm{negative}\kern0.5em \mathrm{tubes}\kern0.5em \times \kern0.5em \mathrm{ml}\kern0.5em \mathrm{sample}\kern0.5em \mathrm{in}\kern0.5em \mathrm{all}\kern0.5em \mathrm{tubes}\right)}} $$

Standard deviations can also be calculated for MPN estimates using the equation below:

2 $$ \pm 0.58\sqrt{\frac{{ \log}_{10}a}{n}} $$

where *a* = dilution ratio and *n* = number of tubes (wells) per dilution.

Control plates were included alongside each test (as described in Thouand et al. 1995), including plates containing inoculum but no 4-nitrophenol and plates with 4-nitrophenol but no inoculum. The variability within each 96-well plate was studied by comparing the frequency of degradation across 12 replicate columns of eight wells on each plate. The variability and probability of biodegradation provide an indication of the reliability of the test method.

### Total cell counts

Inocula total cell counts were determined by epifluorescence microscopy following staining with the nucleic acid-binding fluorochrome 4′,6-diamidino-2-phenylindole (DAPI, Sigma-Aldrich, UK) as previously described (Davenport et al. 2000). Samples were serially diluted using Milli-Q water (Eppendorf AG, Hamburg, Germany) containing DAPI at a final concentration of 33 μg/ml. The samples were incubated for 30 min at room temperature in the dark. Before filtering through a polycarbonate nucleopore filter (pore size 0.2 μm, Millipore, USA), 30 μl of sample was then mixed with 70 μl of sterile distilled water. Once dry, the filters were placed onto a drop of anti-fadant (Citifluor Ltd., Canterbury, UK) on the surface of a standard glass microscope slide. A second drop of anti-fadant was then added on top of the filter, followed by a glass cover slip which was sealed to the slide using nail varnish. Slides were viewed using an epifluorescence microscope (Olympus BX-40) at ×100 magnification using an oil immersion lens under UV light. Twenty fields of view were sampled randomly, and the average number of cells per millilitre was calculated using the following equation:

3 $$ \mathrm{Total}\kern0.5em \mathrm{number}\kern0.5em \mathrm{of}\kern0.5em \mathrm{cells}/\mathrm{ml}=\frac{\mathrm{Average}\kern0.5em \mathrm{number}\kern0.5em \mathrm{of}\kern0.5em \mathrm{cells}\kern0.5em \mathrm{per}\kern0.5em \mathrm{FOV}\times \kern0.5em \mathrm{Area}\kern0.5em \mathrm{of}\kern0.5em \mathrm{filter}}{\mathrm{Area}\kern0.5em \mathrm{of}\kern0.5em \mathrm{FOV}\times \kern0.5em \mathrm{Volume}\kern0.5em \mathrm{Applied}\kern0.5em \times \kern0.5em \mathrm{Original}\kern0.5em \mathrm{sample}\kern0.5em \mathrm{volume}} $$

### Bacterial community characterisation

Total DNA was extracted from 250 μl of each inocula and selected post-degradation test wells using the FastDNA Spin Kit for Soil (MP Biomedicals, Cambridge, UK) and Ribolyser (Hybaid Ltd., Middlesex, UK) according to the manufacturer’s guidelines. Fragments of the bacterial 16S rRNA gene were amplified by polymerase chain reaction (PCR) using the primers (2 and 3) and conditions specified by Muyzer et al. (1993), targeting conserved regions of the gene The reactions were carried out in 200-μl PCR tubes, containing 1 μl template DNA, 10 pmol (in 1 μl) of each primer and 47 μl of MegaMix-Blue PCR-ready mix (Microzone, Haywards Heath, UK). PCR was performed using a thermal cycler (Thermo, USA).

The community fingerprint method, denaturing gradient gel electrophoresis (DGGE), was used to evaluate the bacterial community diversity, the structure and the identity of selected predominant taxa. This method separates amplified DNA fragments of the same size according to differences in their sequence in a gel containing a gradient of denaturants. This results in a banding pattern (see Fig. 2) representative of the predominant bacterial community in the original sample, where each band represents a putatively different sequence or operational taxonomic units (OTUs). Polyacrylamide gels (10 % polyacrylamide, 0.75-mm thick, 16 by 16 cm) were run in 1× TAE buffer (40 mM Tris–acetate, 1 mM EDTA, pH 8.3) on the D-code system (Bio-Rad, Hercules, CA, USA). A gradient of 30–55 % denaturant (where 100 % denaturant contains 7 M urea plus 40 % *v*/*v* formamide in 1× TAE) was used. Gels were run at 60 °C for 900 V h (approximately 4.5 h at a constant 200 V), then stained for 30 min using SYBR Gold (Sigma, Poole, UK, diluted to 1:10,000 in 1× TAE). Stained gels were viewed and imaged using a Fluor-S gel documentation system (Bio-Rad, Hercules, CA, USA).

To determine the sequence identity of DNA found in DGGE bands, bands were excised by stabbing with a 10-μl disposable pipette tip. The excised gel fragment was then transferred to 50 μl TE buffer, left to elute overnight at 4 °C, re-amplified by PCR using the original primers and then purified using a PCR purification kit (QIAGEN Ltd., Crawley, UK). The purified DNA fragments were sequenced using primer 2 and the BigDye Terminator (v3.1) Cycle Sequencing Kit (Applied Biosystems, Foster City, CA, USA) on an Applied Biosystems’ ABI PRISM 377 DNA Sequencer (Proteomics and Molecular Biology Unit, University of Newcastle upon Tyne). The identity of the nearest matching sequence for each band was determined using the FASTA search in the publicly available EMBL nucleotide sequence database (Pearson 1990).

DGGE gel images were analysed using BioNumerics image analysis and database software (Applied Maths, Austin, USA). This allowed the normalisation of the position of bands relative to a standard marker included in each gel, which contained fragments that produced a known distinctive pattern of bands, thereby allowing the valid comparison of similarity in community fingerprints between multiple gels. Band presence/absence data were used to compare pairwise similarities between the predominant bacterial communities in each sample using the Dice coefficient in the BioNumerics software:

4 $$ \mathrm{Dice}\kern0.5em \mathrm{similaritry}\kern0.5em \mathrm{coefficient}=\frac{\left.2\right|A\cap \left.B\right|}{\left|A\right|+\left|B\right|} $$

where *A* and *B* represent two different sample sets.

The resulting similarity matrix was used to bin similarity values within (replicates) and between samples, in order to perform an analysis of variance (ANOVA). Diversity indices were based on band number and/or using band height as a proxy for abundance. Band richness was used as a proxy for OTU (i.e. taxa or ‘species’) richness (*S*). The Shannon diversity index was calculated as follows (Legendre and Legendre 1998):

5 $$ \mathrm{H}{\prime}_{\max }={\displaystyle {\sum}_{i=1}^S\mathrm{Pi}\kern0.5em  \log \kern0.5em \mathrm{Pi}} $$

6 $$ \mathrm{H}{\prime}_{\max }={\displaystyle {\sum}_{i=1}^S\frac{1}{\mathrm{s}} \ln \frac{1}{\mathrm{s}}} $$

where S = number of species, Pi = relative frequency of observations

The evenness of bacterial communities was calculated using Pielou’s *J*′ index of evenness (Pielou 1975):

7 $$ J\prime =\frac{H\prime }{H{\prime}_{\max }} $$

where *H*′ = number derived from the Shannon diversity index (Eq. 5) and *H*′_max_ = maximum value of *H*′ (Eq. 6).

Statistical analyses were also performed on RBT outcomes. For statistical analyses, all data were tested for normality and non-normal data were either transformed using the Box-Cox transformation or the non-parametric Kruskal–Wallis test was performed, using Minitab (Minitab Ltd., Coventry, UK).

## Results

### The effect of inocula preparation methods on detectable bacterial diversity and cell concentration

#### Unprocessed activated sludge

The sample sites were chosen for putative differences in their bacterial diversity, which were selected on the basis of sludge age. Indeed, Tudhoe Mill WWTP, which had a longer sludge age, had significantly greater detectable OTU richness (28) and evenness (0.72) than Sedgeletch WWTP (20 and 0.62, respectively; *P* < 0.01 and *P* < 0.04, ANOVA, for richness and evenness, respectively; Table 2). Dilution of these inocula resulted in slight, but statistically insignificant, changes in both band richness and evenness. There was also little difference in the bacterial biomass concentration of both WWTPs measured as either total suspended solids or total cell counts (Table 2).

#### The supernatant of settled activated sludge

**Fig. 1:**
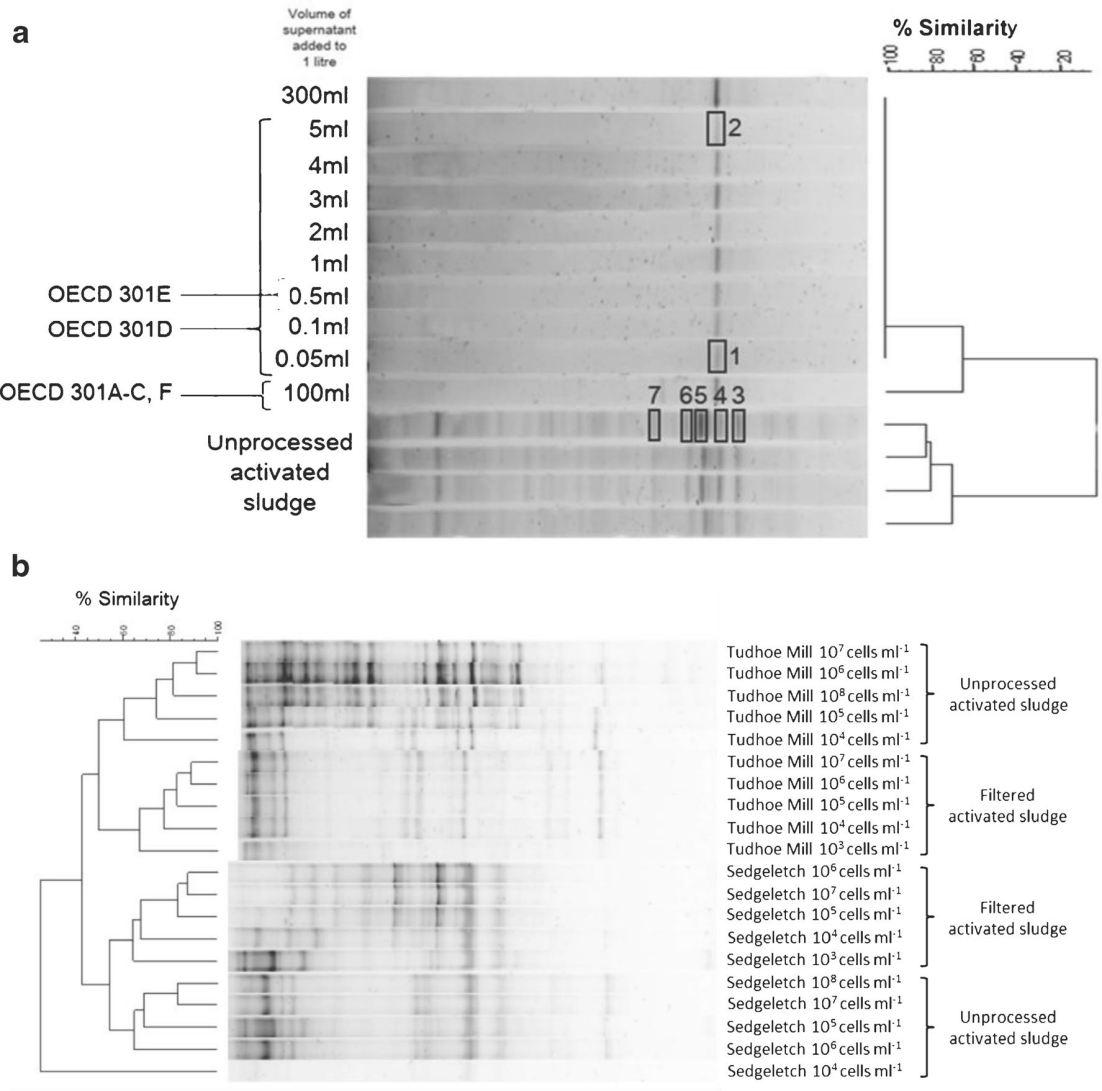
DGGE gels showing differences in bacterial band richness and community structure with their respective dendrograms after cluster analysis based on the community similarity values for **a** the supernatant of settled activated sludge samples and the original unprocessed sample (the volumes used in current standard OECD tests are *marked* and the *boxes* indicate those bands that were excised and sequenced, Table S1) and **b** between filtered activated sludge samples and their unprocessed counterparts for samples from Tudhoe Mill and Sedgeletch wastewater treatment plants

Preparing inocula from the supernatant of settled activated sludge (method i) resulted in a significant reduction in the detectable bacterial diversity, measured as OTU richness, when compared to unprocessed activated sludge (method iii, *P* < 0.001, ANOVA). Only a single common DGGE band was found in all dilutions of inocula prepared in this way, which was absent from the unprocessed activated sludge that had a mean OTU richness of 26 (Fig. 1). The sequence from the single common band (e.g. bands 1 and 2, Fig. 1) was found to be closely related to members of the *Enterobacteriaceae* family (Table S1; supporting information (SI), 100 % similarity), many members of which are a normal part of the gut flora (Sanderson 1976). Band 5, which migrated to a different position on the gel (and had a different sequence to bands 1 and 2), was also related to the same nearest matching sequences although at a lower similarity (Table S1; SI, 95.5 %).

#### The filtrate from filtered activated sludge

**Fig. 2:**
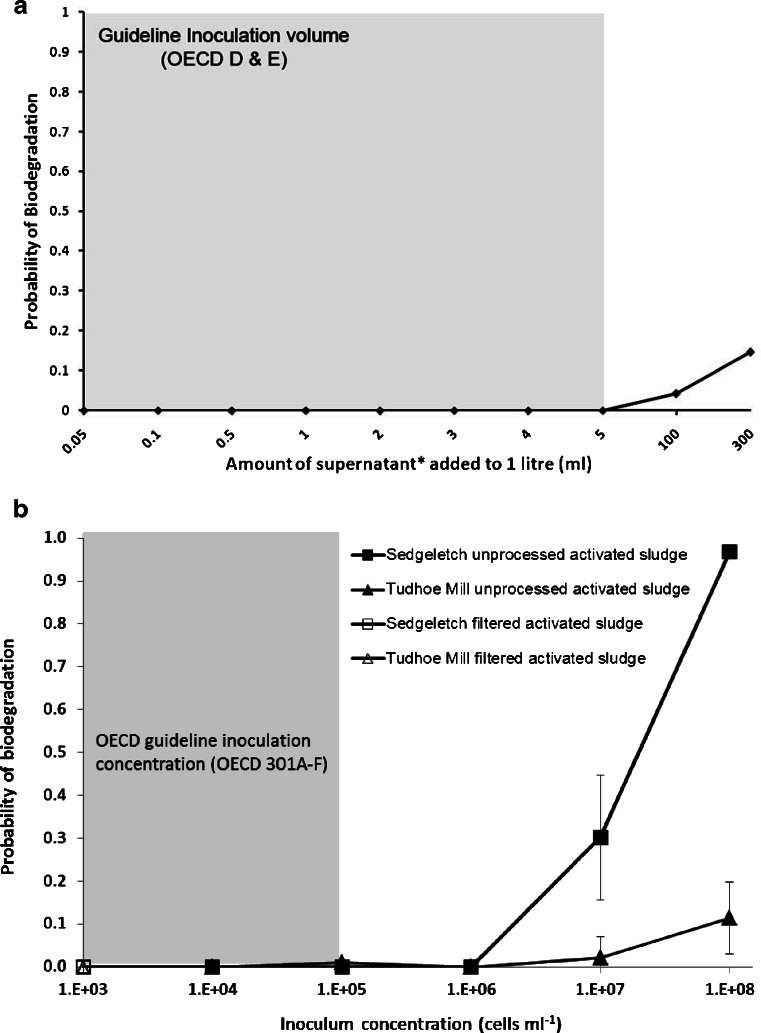
The probability of biodegradation of 4-nitrophenol as a function of cell concentration for inocula prepared according to the international standard OECD guidelines for biodegradation, **a** from the supernatant of settled activated sludge and **b** from filtered activated sludge samples together with those from their unprocessed counterparts. The cell concentrations/volumes used in current standard OECD biodegradation tests are shaded in *grey* and indicated by *text*. *Error bars* indicate standard deviation

Filtering activated sludge reduced the concentration of bacteria by more than 97 % in comparison with their unprocessed counterparts, when measured as total suspended solids (TSS, Table 2) and total cell counts (TCC, Table 2). However, in contrast to the supernatant from settled activated sludge, there were no significant different reductions in the OTU richness after filtering activated sludge (method ii) samples from either Sedgeletch WWTP (*P* = 0.30, ANOVA) or Tudhoe Mill WWTP (*P* = 0.15, ANOVA; Table 2), although there was a distinct shift in community structure (Fig. 2). In addition, there was a reduction in community evenness (Kruskal–Wallis, *P* = 0.03) after filtration for Tudhoe Mill, but not for Sedgeletch, samples (*P* = 0.94, ANOVA; Table 2). Dilution resulted in greater reductions in OTU richness and greater changes in evenness in filtrate samples than unprocessed activated sludge for both Tudhoe Mill and Sedgeletch samples (data not shown). Together, these results demonstrate that filtration leads to an inoculum that is less diverse than the unprocessed activated sludge. The average similarity (Dice coefficient, number of shared bands) between filtered and unprocessed activated sludge samples was 7.68 %, and all samples were dominated by a few highly dominant OTUs (Fig. 2). Filtering the activated sludge excluded on average 58 % of OTUs detected in the activated sludge flocs, and 35 % of those OTUs detected in the filtrate were undetected in the samples from which they were derived (data not shown).

### The effect of inocula preparation methods on the probability of biodegradation

All HT-BSTs inoculated at concentrations recommended by the international standard OECD guidelines for RBTs (Table 1; Fig. 2) failed to biodegrade 4-nitrophenol (mean probability of less than 0.05). Of the samples processed by filtration or settling, the maximum mean probability of biodegradation was 0.14 when using an inoculum 300 times more concentrated than that recommended by the OECD for this method of preparation (OECD 301 D; Fig. 1; 300 ml supernatant per litre) (OECD 1992b). The probability of 4-nitrophenol biodegradation was highly variable for those inocula concentrations showing degradation, with a coefficient of variation of 115 and 148 %, and lower 95 % confidence limits close to zero (0.0396 and 0.0026) for 300 and 100 ml of inocula, respectively, and mean probabilities that were statistically indistinguishable from zero (*P* > 0.05, one tailed *t* test).

Biodegradation was only observed in those HT-BSTs inoculated with unprocessed activated sludge at concentrations that far exceeded those recommended by the OECD guidelines (Table 1). Interestingly, inocula derived from Sedgeletch WWTP, which had the lowest detectable bacterial diversity, had a higher probability of 4-nitrophenol biodegradation, 0.97, than the inocula derived from Tudhoe Mill WWTP, 0.11, albeit at concentrations 3 orders of magnitude greater than those recommended by the OECD guidelines (Table 1). When calculated as specific degrader abundances, activated sludge from Sedgeletch WWTP had significantly more 4-nitrophenol degraders than Tudhoe Mill WWTP (*P* < 0.001, ANOVA).

For both inoculum sources, the observed changes in community structure for unprocessed activated sludge at the different cell concentrations did not directly coincide with the changes in the probability of 4-nitrophenol biodegradation. These results indicate that the degrading communities were a minor component of the overall bacterial community and were below the detection threshold of DGGE; the abundance of 4-nitrophenol degraders was calculated as 5.80 × 10^4^ cells ml^−1^ for Sedgeletch WWTP (0.000007 % from MPN and TCC data) and 4.38 × 10^2^ cells ml^−1^ for Tudhoe Mill WWTP (0.0000003 % from MPN and TCC data).

## Discussion

This study is the first to systematically evaluate the effect preparation methods used for the majority (six out of seven) of standard regulatory screening tests have on the detectable bacterial diversity, the concentration of bacteria in inocula and the probability of biodegradation. The process of settling and filtering activated sludge resulted in distinct shifts in the bacterial community composition compared to their unprocessed counterparts. For many of the samples, these preparations further resulted in statistically significant reductions in measures of the detectable bacterial diversity. The most drastic reductions were observed for the preparation of inocula using the supernatant of settled activated sludge. In this sample, a single dominant taxon was detected in all dilutions, which was closely related to the members of the *Enterobacteriaceae* family. This particular taxon was absent from the detectable diversity of the activated sludge, indicating that it may represent a planktonic bacterium that was either absent or rare in activated sludge flocs. The low number of shared OTUs between the filtrate of filtered activated sludge and unprocessed sludge also suggests that planktonic, non-floc bacteria were selected by this method of preparation. It must be noted that co-migration and multiple gene copies can influence the number of bands (OTUs) visualised by DGGE (Muyzer et al. 1993; Klappenbach et al. 2001). Criticism often levelled the use of DGGE because of its low resolving power in terms of diversity. Indeed, it is estimated that the detection threshold for DGGE is about 1 % (10^7^ cells ml^−1^), and therefore, only the most abundant taxa in any given sample can be resolved with many rare taxa excluded (Akarsubasi et al. 2009; Woodcock et al. 2006). However, some of the differences reported here are stark and statistically significant. Others were measured as evenness, which may provide a better indication of relative differences in diversity as it depends on the taxa abundance distribution rather than on the total number of OTUs per se and can therefore be meaningfully applied to abundant taxa. Furthermore, recent studies using next-generation sequencing suggest that as few as 10–100 sequences can distinguish the same significant differences in community structure observed using tens of thousands of sequences (Caporaso et al. 2012; Kuczynski et al. 2010). Thus, the differences in bacterial community structure and diversity of the abundant taxa detected in this study are significant.

In this study, the reduced detectable bacterial diversities of settled supernatant and the filtrate of activated sludge were associated with a lack of 4-nitrophenol biodegradation even when such inocula were 1,000 times more concentrated (10^8^ cells ml^−1^) than those stipulated in the OECD guidelines (OECD 1992b). Thouand et al. (1995) reported a probability of 4-nitrophenol biodegradation of 1 at the same inoculum concentration for six samples of activated sludge from a single municipal WWTP in France when using a similar filtration method. This discrepancy may be, in part, due to a small difference in the preparation method, as Thouand et al. (1995) ultrasonicated their activated sludge samples prior to filtration.

In contrast, unprocessed activated sludge samples with greater detectable diversities demonstrated a probability, and hence reliability, of 4-nitrophenol biodegradation of between 14 and 97 % in a 28-day test, but only at concentrations that exceeded the OECD guideline values by 1,000 times (10^8^ cells ml^−1^). There were differences in the probability of 4-nitrophenol biodegradation for the two inocula sources used in this study. The inoculum with the lowest expected and observed bacterial diversity, Sedgeletch, was associated with a greater probability of 4-nitrophenol degradation. This outcome could arise for several reasons: (1) there is little or no relationship between biodegradation potential and bacterial diversity (e.g. Griffiths et al. 2001), (2) the detectable diversity inaccurately reflects the true diversity and rarer taxa below the detection threshold of DGGE were more diverse in Sedgeletch samples (Akarsubasi et al. 2009), (3) the biodegradation outcome reflects the success of fast-growing bacteria that would be more numerically dominant in Sedgeletch, a WWTP with low sludge age (Smalla et al. 1998; Van Ginkel et al. 1995; Vazquez-Rodriguez et al. 2000), (4) the differences are a result of natural variation in population abundances of 4-nitrophenol-degrading bacteria or (5) a combination of the factors above. The absolute total number of bacteria in typical OECD test would exceed that used in this study as the HT-BSTs were conducted in microlitre rather than millilitre volumes to achieve greater replication and statistical power. However, chemical and inocula concentrations similar to those in OECD tests were maintained, so the food-to-microorganism ratio would have been equivalent for the lower inocula concentrations. Even if the total number of bacteria to which a chemical is exposed is critical, the mean probability of 4-nitrophenol biodegradation was low (<0.05) and highly variable (coefficient of variation of 115 %) at inocula concentrations where the total numbers of cells were equivalent to those in OECD tests (i.e. approximately 10^8^ cells ml^−1^ in the HT-BSTs).

By conducting highly replicated tests with multiple inocula concentrations sourced from different sites for a single compound (the equivalent of about 3,600 individual biodegradation tests), a value for the reliability of 4-nitrophenol biodegradation has been made possible. The results presented here are consistent with previous studies that have shown 4-nitrophenol biodegradation to be highly variable in RBTs (Thouand et al. 1995; IPCS 2000), with a median half-life of 2.5 days in non-standard freshwater tests, but with a range of 1.3–77 days (Comber and Holt 2010). It is currently classified as inherently biodegradable by IPCS (2000). Together, these data demonstrate that (1) there are large variations within and between current RBTs (Blok and Booy 1984; Gerike and Fischer 1979, 1981; Nyholm et al. 1984; Painter 1995; Thouand et al. 1995) and (2) greater inocula biomass concentrations reduce variability and/or increase the likelihood of biodegradation for a given compound (Blok and Booy 1984; Nishino and Spain 1993; Nyholm et al. 1984; Painter 1995; Ramadan et al. 1990; Thouand et al. 1995; Vazquez-Rodriguez et al. 2000). In previous studies, some of this variation has been attributed to the source or quality of the inoculum (e.g. Blok and Booy 1984). This study has, for the first time, been able to quantify this phenomenon, showing that the preparation procedure can significantly alter the microbial abundance, community structure and its diversity compared to the microbial population in the original activated sludge sample from which the inoculum was prepared.

In terms of assessment and terminology, biodegradability and persistence are often viewed as intrinsic chemical properties, which are not. These are complex process properties that in the environment are influenced by many different factors (ECETOC 2003), of which biodegradation is one of the most important and least understood. In chemical regulation, biodegradability and persistence are assessed in biodegradation tests. There are many aspects of these tests that have limitations (see Painter 1995 for a review), but understanding how bacteria vary between inocula is a critical part of addressing the uncertainties associated with biodegradation. Standard international biodegradation screening tests, exemplified by the OECD 301 series, were originally designed to screen out chemicals that would undergo rapid ultimate biodegradation in the environment. Preparation methods, therefore, intentionally reduce the concentration of bacteria in the inocula used (and thus their diversity) in order to increase test stringency. However, while regulatory pressures have changed to identifying persistent chemicals, the internationally accepted standards for determining the biodegradability of a chemical in the environment have not evolved at the same pace.

Biodegradation tests are largely performed once, using a single low-concentration inoculum for a given chemical. We have shown that specific degraders for a chemical might be found in the environment, but are rarely sampled due to the small inoculum sample size used in RBTs. Inocula cell concentrations used in current RBTs are extremely low, 10^5^–10^8^ cells l^−1^, in comparison to the activated sludge samples from which they are derived, 10^10^–10^12^ cells l^−1^, and lower than those in surface waters (lakes, rivers, seas; 10^9^ cells l^−1^). The bacterial diversity between different environmental compartments for different sample sizes is known to vary widely (Curtis et al. 2002).

The variability of RBT results has led to widespread calls for the enhancement of inocula, using increased cell concentration and inoculum preparation techniques (Blok and Booy 1984; Painter 1995; Thouand et al. 1995; Vazquez-Rodriguez et al. 2000). Similar guidance is provided for those performing screening tests for persistence assessments under REACH (ECHA 2008), and the results presented here support the use of environmentally relevant concentrations of inocula to improve the reliability of tests.

Given the inter- and intra-test variation that is observed for the same chemical in different studies, these current assessments appear to test the inoculum as much as they test the chemical itself. Notwithstanding the variation in inocula sources, concentrations and different test methods, the current single-inoculum single-test paradigm appears to be flawed. An improved rationale would be to capitalise on variations between biodegradation tests using different inocula at environmentally relevant concentrations, to provide a probabilistic approach on the likelihood that a chemical will meet a specific degrader (Goodhead 2010; Thouand et al. 2011). This approach would be even more powerful if combined with growth rate data (Blok and Booy 1984).

## Conclusions

In this study, we have shown that the sample preparation of activated sludge inocula advocated for standard international biodegradation tests drastically reduces not only the number of bacteria but also the overall detectable diversity of bacteria in the inocula compared to the original samples. These factors led to increased variability between biodegradation tests and therefore a reduced reliability of the tests to identify those chemicals that are likely to be persistent in the environment. We propose that this inherent variation can be beneficially used to determine a probabilistic likelihood of a chemical encountering a specific degrader in the environment.

## Electronic supplementary material

Below is the link to the electronic supplementary material.

ESM 1 (PDF 103 kb)
