# Supplementary material for: Standard inocula preparations reduce the bacterial diversity and reliability of regulatory biodegradation tests
Source: Environ Sci Pollut Res Int. 2013 Sep 17;21(16):9511–21. doi: 10.1007/s11356-013-2064-4 (PMC4133024; doi:10.1007/s11356-013-2064-4)
Supplement: Supplementary file 1 — (PDF 103 kb) [file 11356_2013_2064_MOESM1_ESM.pdf]

Supporting Information:

Standard Inocula Preparations Reduce the Bacterial Diversity and Reliability of Regulatory Biodegradation Tests

Andrew K Goodhead · Ian M Head · Jason R Snape · Russell J. Davenport

A.K. Goodhead

Sartorius Mechatronics UK Limited, Longmead Business Centre, Blenheim Road, Epsom, Surrey, KT19 9QQ, UK

I.M. Head • R. J. Davenport (✉)

School of Civil Engineering and Geosciences, Cassie Building, Newcastle University, Newcastle upon Tyne, NE1 7RU, UK.

e-mail: russell.davenport@ncl.ac.uk

J. R. Snape

Astrazeneca UK Ltd, Brixham Environmental Laboratory, Freshwater Quarry, Brixham, Devon, TQ5 8BA, UK.

Supporting information is provided as a compilation of closest relatives and their similarities with the sequence of dominant bands excised from the DGGE gels.

Material		Page
Table S1	Nearest neighbour and RDP classification of excised band sequences from the supernatant of activated sludge and its unprocessed counterpart.	S2

Table S1. Nearest neighbour and RDP classification of excised band sequences from the supernatant of activated sludge and its unprocessed counterpart.

Band[§]	Closest relative and accession number	RDP classification*	Percent identity	Origin	Reference
1,2,5[a]	*Erwinia persicinus* (AM184098)	family *Enterobacteriaceae*	100.0, [a]95.5	Processed vegetables	[46]Randazzo et al. (2009)
	Klebsiella sp. HBB9 (EU074057)	family *Enterobacteriaceae*	100.0, [a]95.5	Grapevine juice	
	Enterobacter hormaethei subsp. Steigerwaltii (AJ853890)	family *Enterobacteriaceae*	100.0, [a]95.5	Surgical skin wound	[47]Hoffmann, H. (2005)
	Sludge granule bacterium (EF190022)	family *Enterobacteriaceae*	100.0, [a]95.5	Sludge granules	[48]Adav et al., (2007)
3	*Roseobacter sp*. TKW (DQ479380)	class *Betaproteobacteria*	81.5	Kuwaiti coastal water	[49]Al-Sarawi et al. (2008)
4	*Microscilla sp*. MED571 (EU253600)	no root	49.3	NW Mediterranean surface water	
6	Uncultured bacterium clone FCPS400 (EF516835)	class *Betaproteobacteria*	78	Californian grassland soil	[50]Cruz-Martinez et al (2009)
7	Uncultured *Dechloromonas sp*. clone KS-488 (EU809559)	phylum *Proteobacteria*	81.4	Indian soil	[51]Srinivas et al. (2011)
8	*Flavobacterium gelidacus* type strain LMG21477 (AJ440996)	genus *Flavobacterium*	71.4	Antarctic microbial mat	[52]van Trappen et al. (2002)

[§]Band numbers correspond to those excised from the DGGE gel (Figure 1); [a] percentage identity for band 5 only; * using the Ribosomal Database Project (RDP) Classifier tool.
